# Psychometric Properties of the Persian Version of the Academic Anxiety Scale (AAS)

**DOI:** 10.1002/brb3.71011

**Published:** 2025-11-27

**Authors:** Faezeh Peimanpak, Simin Hosseinian, Abbas Abdollahi

**Affiliations:** ^1^ Department of Counseling, Faculty of Education and Psychology Alzahra University Tehran Iran

**Keywords:** academic anxiety, psychometrics, reliability, student, validity

## Abstract

**Introduction:**

Anxiety is one of the basic problems of students that can cause significant psychological and emotional harm to students. The Academic anxiety scale (AAS) is a self‐report scale for measuring academic anxiety that measures the tendency of individuals to experience test anxiety with 11 items. The present study aimed to investigate the psychometric properties of the Persian version of the AAS in an Iranian sample.

**Methods:**

The research method employed a descriptive approach, and the research sample consisted of 690 students (356 girls and 334 boys) from the second secondary school level in Tehran schools during the academic year 2024–2025. The students were selected non‐randomly and responded to the research instrument.

**Result:**

To examine the reliability and internal consistency of the AAS, CR and Cronbach's alpha coefficient were used, and the results showed that this scale has appropriate internal consistency and CR. Also, the face validity and content validity of the questionnaire were evaluated and confirmed. Construct validity was also determined and confirmed using confirmatory factor analysis (CFA) for this questionnaire. To evaluate convergent validity (CV), the CR and the average variance extracted (AVE) method were used, and the correlation of the AAS with the test anxiety questionnaire, big five personality questionnaire, and beck depression inventory was examined. The results showed that the AAS's CR was 0.887. Correlation matrix analysis of the study variables showed that the AAS has a significant correlation with Test anxiety (*r* = 0.686, *p* < 0.01), the beck depression (*r* = 0.755, *p* < 0.01), and the big five personality factors (neuroticism (*r* = 0.369, *p* < 0.01), agreeableness (*r* = –0.341, *p* < 0.01), conscientiousness (*r* = –0.119, *p* < 0.01), extraversion (*r* = –0.267, *p* < 0.01), openness (*r* = –0.137, *p* < 0.01)).

**Conclusion:**

Overall, the results of the study showed that this scale has favorable psychometric properties in the Iranian sample and can be used with confidence in Iranian samples.This study evaluates the psychometric properties of the Persian version of the AAS among Iranian high school students. A sample of 690 students participated. Results from reliability and validity analyses, including Cronbach's alpha and CFA, confirmed the scale's strong internal consistency, construct validity, and CV. The AAS showed significant correlations with test anxiety, depression, and big five personality traits. Findings suggest the AAS is a reliable tool for assessing academic anxiety in Iranian adolescents.

## Graphical abstract

This study evaluates the psychometric properties of the Persian version of the Academic Anxiety Scale (AAS) among Iranian high school students. A sample of 690 students participated. Results from reliability and validity analyses, including Cronbach's alpha and confirmatory factor analysis, confirmed the scale's strong internal consistency, construct validity, and convergent validity. The AAS showed significant correlations with test anxiety, depression, and Big Five personality traits. Findings suggest the AAS is a reliable tool for assessing academic anxiety in Iranian adolescents.

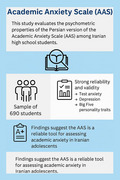

## 1 Introduction

Academic anxiety is a broad and comprehensive concept that focuses on the non‐clinical anxieties that students experience in various educational contexts. This concept refers to the experience of negative emotional feelings that arise in response to academic stressors and is one of the most significant types of anxiety during adolescence, threatening the mental health of students and adversely affecting their efficacy, the flourishing of their talents, and the formation of their personality and social identity (Ahmadi et al., 2012). Academic anxiety, as a wide structure, encompasses anxieties related to educational activities and is part of a hierarchical structure. This means that general anxieties experienced in all aspects of life influence academic anxiety, and in turn, academic anxiety dominates more specific anxieties, such as exam anxiety (Cassady et al. 2019). Understanding this hierarchical structure allows us to consider academic anxiety as a comprehensive and general concept that includes various types of educational anxieties (Cassady 2022).

The emergence of anxiety in any individual is shaped by the interaction between environmental stimuli (such as academic expectations) and personal factors (including the individual's propensity for anxiety, feelings of efficacy, and past experiences), leading to an assessment of the level of threat posed by tasks and the individual's ability to manage these threats (Cassady 2022; Putwain and Daly 2014). When the evaluation of a situation is based on the student's abilities, the task is typically identified as a challenge that is not threatening. However, if the evaluation indicates that situational pressures and personal resources for performing the task exceed the student's control, this situation is perceived as a threat, increasing the likelihood of academic anxiety (Keeley et al. 2008; Putwain and Symes, 2011; Poutouin et al., 2012). Fear of performing worse than peers, concerns about managing responsibilities, and experiencing stress in classrooms are among the key elements of this type of anxiety. The structure of academic anxiety is primarily defined as a generalized form of various types of anxiety in educational environments (Cassady et al. 2019), encompassing worry, irritability, and nervous feelings related to educational tasks and progress in the educational path, as well as perceived threats due to the presence of stressors in a context related to any educational task. It includes three main components: cognitive, emotional, and behavioral, emphasizing the social aspect when an individual's performance is evaluated by others (Bülbül and Odaci 2023).

In general, interpretations related to the experience of anxiety for students typically focus on four broad areas. The first area centers on self‐assessment of cognitive limitations or inadequate skills for task management. These cognitive limitations (or simply the perception of limitations) may be accurate assessments of ability and readiness or misinterpretations of the requirements facing the student or their preparedness to meet these goals (Cassady 2010; Poutouin and Aviard 2018; Sommer and Arendasy 2014). The second area includes social pressures arising from the expressed or implicit expectations of significant individuals in the student's life and comparisons with peers (Lowe and Lee 2008; Van der Embse and Witmer 2014). In a related vein, the third cause of experiencing academic anxiety is the acceptance of unrealistic personal standards that are not realistically achievable (such as perfectionism) (Stober 2018; Burkas and Kurt 2021). Finally, the fourth influential factor in the experience of academic anxiety is an unusually high level of pressure or stress in specific temporal or situational conditions. That is, individuals who may not generally be prone to experiencing high levels of academic anxiety can experience significant anxiety in isolated environments, such as high‐stakes assessments, performance in front of a group, or when a stressful or threatening academic event occurs unexpectedly (Poutouin and Van der Embse 2018).

Both positive and negative effects on students' academic performance can result from exam and homework anxiety. Students who are under pressure to perform well academically may become more motivated, focused, and put forth more effort as a result of approaching tests and assignments. But for others, this anxiety can become unbearable, resulting in excessive worry, procrastination, or trouble concentrating before tests, all of which can hinder learning. Additionally, it may result in issues during the test itself, including trouble concentrating or forgetting crucial details, a sense of unpreparedness, and the possibility of subpar performance. This type of anxiety is considered an unpleasant emotional reaction to educational situations and is among the common disorders among Iranian adolescents, observed in nearly all Iranian students to varying degrees throughout their lives (Saye Miiri et al. 2021). Research indicates that between 10% to 30% of Iranian students face severe types of this anxiety (Jiriayi Sharahi and Hashemi Moghadam 2021). Therefore, identifying and developing various tools to measure this phenomenon is deemed essential. Considering that the currently standardized scales for measuring academic anxiety in Iran are based on past data and conditions and may no longer reflect the existing situation and needs, validating a new scale to align with temporal and cultural changes seems necessary.

The AAS (Cassady 2022; Cassady et al. 2019) is a measure of the construct academic anxiety, which is a generalized representation of anxieties experienced by learners in educational settings. Cassady et al. (2019) in the initial validation of this tool demonstrated that academic anxiety is strongly related to test anxiety and general neuroticism, predicts depression symptoms, and has a weak to moderate association with college GPA. Cebu et al. (2023) examined the relationship between demographic factors and academic anxiety among Filipino senior high school students using the AAS with 270 respondents. Findings revealed significant differences in academic anxiety across age, gender, and grade level, highlighting the need for supportive psychological interventions in educational settings. In another study, Finch et al. (2024) used latent class analyses to identify severity levels for learners completing the AAS, four classes of learners were characterized by levels of academic anxiety: Non‐anxious, mild academic anxiety, moderate academic anxiety, and high academic anxiety. Nevertheless, this tool has not yet been utilized within the Iranian community, highlighting the necessity for further investigation in this area. In this regard, the present study examines the validity of the AAS to provide more accurate assessments of academic anxiety in the current educational context of Iran.

## 2 Materials and Methods

### 2.1 Participants

Participants ranged from 16 to 18 years of age, and the data collection used a non‐random sample technique. In total, 690 secondary school students from Tehran participated by filling in an online questionnaire on Porsline. The sample was composed of 356 women (51.6%) and 334 men (48.4). The mean age of participants was 16.67 years, and the standard deviation was 0.76 years. Out of this number, 353 students (51.2%) were in the tenth grade, 210 students (30.4%) were in the eleventh grade, and 127 students (18.4%) were in the twelfth grade, with an average of the last academic grade of 18.04. The approximate time required to complete the questionnaires was 10 min, The questionnaires on the Porsline website were completed after each participant provided informed consent. This estimated duration was based on the researchers’ assessment, considering the self‐report nature and clarity of the items. This scale gathered participants’ demographic information, including age, gender, the last grade point average, and level of education, in the December 2024‐ March 2025 interval according to the test administration guidelines of the Research Ethics Committee of Alzahra University (IR.ALZAHRA.REC.1403.063).

### 2.2 Instruments

#### 2.2.1 AAS

This scale was designed by Cassady et al. (2019) to measure academic anxiety in students. It consists of 11 questions to which participants respond on a 4‐point Likert scale (1 = does not apply to me at all, 2 = somewhat applies to me, 3 = applies to me, and 4 = very much applies to me). The main objective of this scale was to create a broad and simple picture of the anxieties experienced in educational environments. The psychometric properties of the AAS were reported to be adequate in the original study (Cassady et al. 2019). However, this scale has not yet been used in Iran, and this research will determine its psychometric characteristics. The Cronbach's alpha coefficient for this scale in the present study was found to be 0.88.

#### 2.2.2 Test Anxiety Scale

To measure different aspects of test anxiety, including stress, cognitive errors, and social humiliation, Friedman and Bendas Jacob (1997) created this 23‐item scale using a four‐point Likert response format. In their study, Friedman and Bendas Jacob found that the subscales measuring stress, social humiliation, and cognitive errors had alpha coefficients of 0.86, 0.85, and 0.81, respectively. The complete scale had an overall reliability of 0.91. Correlation coefficients of 0.84 for boys and 0.82 for girls were obtained by comparing the scale to the Spielberger test anxiety scale in order to verify its validity. A different study by Ba'ezat et al. (2012) evaluated this scale's psychometric qualities in the context of Iran, revealing that Cronbach's alpha coefficients for each of its components ranged from 0.83 to 0.90. Furthermore, exploratory factor analysis showed that every item was correctly connected to the test score overall and had correlations greater than 0.3. The scale's reliability was also assessed by Janabadi and Salarpour (2021), who reported that the subscales had Cronbach's alpha values of 0.88, 0.78, and 0.84, while the entire questionnaire had a reliability of 0.90. In the present investigation, the test anxiety scale's Cronbach's alpha coefficient was found to be between 0 and 90.

#### 2.2.3 Big Five Personality Questionnaire (Short Form)

Based on the results of Khormayi's research (2006), a shorter version of the big five personality questionnaire by Goldberg (1999) was created, which includes 21 items and 5 subscales: Neuroticism (4 items), agreeableness (4 items), conscientiousness (4 items), extraversion (5 items), and openness to experience (4 items). This questionnaire is used to measure the big five personality factors on a five‐point Likert scale from 1 (strongly disagree) to 5 (strongly agree). The psychometric properties of this questionnaire have been examined in Iran, and Khormayi (2006) confirmed the independence of these five factors among students at Shiraz University through factor analysis. The factors explained 11.4%, 8.46%, 8%, 7.73%, and 7.2% of the variance, respectively, and together accounted for 43% of the total variance. In Khormayi and Farmani's study, the reliability of this questionnaire was reported with Cronbach's alpha coefficients ranging approximately from 0.88 for neuroticism to 0.77 for extraversion (Khormayi and Farmani 2014). The validity of the short form of the big five personality questionnaire was confirmed using principal component analysis with varimax rotation, which revealed five distinct factors. The sample adequacy index (KMO = 0.79) and Bartlett's test of sphericity (χ^2^ = 532.3, *p* < 0.001) indicated suitability for factor analysis. Khormayi and Farmani (2014) reported Cronbach's alpha values of 0.83 for neuroticism and agreeableness, 0.81 for conscientiousness, and 0.72 and 0.69 for extraversion and openness, respectively. In the present study, the alpha coefficients were 0.70 (neuroticism), 0.73 (agreeableness), 0.70 (conscientiousness), 0.75 (extraversion), and 0.69 (openness), with an overall reliability of 0.71.

#### 2.2.4 Beck Depression Inventory (BDI)

Using a four‐point Likert scale (from zero to three), this 21‐item questionnaire, created by Beck in 1996, evaluates the degree of depression symptoms in people 13 years of age and older. A score of zero indicates the lowest level and a score of three indicates the highest level of severity of experiencing a depressive symptom. The CV of this questionnaire was established through its simultaneous administration with the Dyce (1996) and the Beck Anxiety Inventory (1993), yielding correlation coefficients of 0.68 and 0.60, respectively, while the reliability of this scale was reported as 0.78 using Cronbach's alpha (Beck et al. 1996). In Iran, Taheri Tanjani et al. (2015) reported an intraclass correlation coefficient of 0.81 and a Cronbach's alpha of 0.93 for this questionnaire. To assess the concurrent validity of the BDI, the general health questionnaire and the depression, anxiety, and stress scale were used. The correlation coefficients of the BDI with each of the depression (0.73), anxiety (0.68), and stress (0.63) subtests of the depression, anxiety, and stress scale indicate that the test has good concurrent validity (Taheri Tanjani et al. 2015). In the present study, the Cronbach's alpha coefficient for this questionnaire was found to be 0.94.

### 2.3 Procedure

The AAS was translated into Persian using Brislin's methodology. First off, a professional psychologist translated the first edition of the AAS from English into Persian. After that, an English specialist who was unfamiliar with the English scale and its sentences back‐translated the synthesized Persian version into English. Thirdly, the original AAS version and the back‐translated version were contrasted. Three experts from the fields of psychiatry, psychology, public health, and clinical practice made up the committee that discussed this comparison. The committee discussed and resolved any discrepancies that were discovered. Finally, the researchers dispersed the scale among 20 people in a convenient manner and eliminated any current lexical ambiguities in order to get feedback from participants, check their perceptions of the items, and rule out any potential deficits (Jones et al. 2001).

Likewise, the SPSS 26 and AMOS 23 software were used to analyze the data and examine the psychometric properties of AAS.

The AAS's face validity was investigated using a qualitative methodology, i.e., determining the degree of difficulty, unsuitability, and ambiguity of sentences as well as the semantic insufficiency of words, which required a team of five experts and faculty. Both quantitative and qualitative analyses of content validity assess how well items address the topic's goal. Five faculties were asked to submit their written corrective opinions for the qualitative portion after carefully reviewing the scale items, taking into account syntax, appropriate wording, the importance and positioning of the questions, and the allotted time for completing the instrument. Following the gathering of expert opinions, the required adjustments were made. The researchers then employed the content validity index (CVI) to make sure the items were properly designed for measuring the content and the content validity ratio (CVR) to ensure the selection of the most important and accurate content. For CVR measurement, 15 specialists were required to score every item on a three‐point Likert scale: not necessary (1), useful but not necessary (2), and necessary (3).

In order to examine the construct validity of AAS, maximum likelihood confirmatory factor analysis was used. According to Kline (2023), items with this feature were excluded from the analysis because their factor loading should be higher than 0.4. Goodness of fitness (GOF) indices were used to examine the model's validity. For a properly fit model, the comparative fit index (CFI), Tucker‐Lewis index (TLI), and incremental fit index (IFI) should be above 0.90. Likewise, the parsimony comparative fit index (PCFI) and parsimony normed fit index (PNFI) should exceed 0.50. Although the Chi‐squared (*x^2^*) index is often used to investigate GOF, *x^2^* is related to the large sample size and degree of freedom and, hence, is not usually confirmed (Kline 2023). For this reason, the root mean square error of approximation (RMSEA) and minimum discrepancy divided by degree of freedom (CMIN/DF) are employed. Thus, if RMSEA is < 0.08 and CMIN/DF is < 3, the model fits acceptably (Kline 2023). In CFA, covariation of errors suggests that there are correlations between the residuals of two indicators that are not accounted for by the latent variables in the model. It is implied that there are common sources of variance between these pairs of observed variables beyond what can be explained by their individual latent factors when modification indices indicate covarying errors. A more accurate depiction of the connections between your measured variables and latent constructs results from allowing these error covariances because doing so enhances the model fit by recognizing and taking these correlations into account (Kline, 2023).

The researchers investigated internal consistency using the Cronbach alpha coefficient and CV and CR using the average variance extracted (AVE). According to Fornell and Larcker (1981), CR values of > 0.60 indicate acceptable internal consistency of a construct. Besides, AVE values of > 0.50 hint CV.

Similar to Cronbach's alpha, CR (also known as CR) is a measure of internal consistency in scale items. One way to conceptualize it is as the sum of the true score variance and the scale score variance. By comparing the amount of shared variance, or covariance, among the items that comprise an instrument to the amount of overall variance, Cronbach's alpha is a method of evaluating reliability. According to the theory, there should be a high degree of covariance between the items in relation to the variance if the instrument is dependable.

## 3 Results

### 3.1 Face Validity

The opinions of experts were applied to the questionnaire as slight modifications.

### 3.2 Content Validity

The item may be retained if the index value is greater than 0.49, considering the number of specialists and the Lawshe table (1975). No items were eliminated because the results demonstrated that all of the items' coefficients ranged from 0.73 to1. Moreover, the overall content validity ratio (CVR) for the AAS was found to be 0.80. Three criteria were also used to evaluate the content validity index (CVI): difficulty, relevance, and ambiguity, using a four‐option Likert scale: unrelated (1), requires serious revision (2), relevant but requires revision (3), and completely relevant (4). This index must have a value greater than 0.79 (Waltz and Bassil 1981). TheCVI items for all three dimensions had values between 0 and 1, indicating that no items needed to be removed or altered. This led to the ambiguity index, relevance dimension, and difficulty dimension all receiving zero points for the CVI of the entire questionnaire. The results show that the AAS has adequate content validity.

### 3.3 Descriptive Statistics

To enhance methodological transparency and support replication, detailed information regarding the assumptions tested for the CFA is provided in the supplementary material. Specifically, the data were screened for univariate normality through skewness and kurtosis values, all of which fell within acceptable ranges (±2). Before data analysis, the skewness and kurtosis indices were used to evaluate the items' normality. According to Tabachnick and Fidell (2019), acceptable skewness and kurtosis values should be within ±2 and ±5, respectively. Multivariate normality was assessed using Mardia's test, confirming that the data met the assumption of multivariate normality. Model identification was thoroughly checked to ensure the model parameters could be reliably estimated. Additionally, multicollinearity among observed variables was evaluated through variance inflation factors (VIFs), all of which were below the recommended threshold of five, indicating no significant multicollinearity issues. These checks provide a robust foundation for the validity of the CFA results (see Table 1).

**TABLE 1:** Descriptive statistics and factor loadings.

Statements	Factor loading	Mean	Standard deviation	Skewness	Kurtosis
1. I often worry that my best performance is not as good as expected in school.	0.58	2.02	0.97	0.72	−0.44
2. I tend to procrastinate on schoolwork because I become anxious.	0.59	1.72	0.95	1.21	0.38
3. I often worry that I am not doing my assignments correctly.	0.64	1.89	0.98	0.84	−0.39
4. I have less confidence in school compared to my classmates.	0.55	1.86	0.98	0.95	−0.14
5. I feel panic when I am in class.	0.68	1.46	0.81	1.87	2.75
6. I tend to find my teachers intimidating.	0.70	1.39	0.83	2.06	3.04
7. Most of my time at school is spent worrying about what is ahead of me.	0.67	1.83	0.94	0.95	−0.03
8. There is something about school that scares me.	0.73	1.51	1.51	1.72	2.28
9. I worry about what my classmates think of my abilities.	0.70	1.75	1.75	1.23	0.37
10. When I have to work on a major class assignment, I often feel sick.	0.53	1.47	1.47	1.88	2.54
11. I have difficulty managing my academic responsibilities.	0.68	1.69	1.69	1.30	0.60

*Note*: Factor loading = standardized factor loading; Mean = average score; SD = standard deviation; Skewness = measure of asymmetry; Kurtosis = measure of peakedness of the distribution.

### 3.4 Construct Validity

Table 1 indicates that all indicators demonstrated adequate loading values, confirming their capacity to reliably measure their corresponding latent constructs. According to Tabachnick and Fidell (2019), factor loadings of 0.71 and above are considered excellent, those ranging from 0.63 to 0.70 are very good, 0.55 to 0.62 are good, 0.45 to 0.54 are fairly good, 0.32 to 0.44 are weak, and values below 0.32 are deemed poor.

Additionally, as shown in Table 2, all fit indices derived from the CFA supported an acceptable model fit to the observed data (χ^2^/df = 3.89, CFI = 0.990, RMSEA = 0.089, GFI = 0.998, TLI = 0.980, IFI = 0.995, NFI = 0.989, RFI = 0.977). However, an evaluation of the modification indices suggested that introducing covariances between the error terms of certain indicators would enhance model fit. Accordingly, the measurement model was modified, resulting in improved fit indices, thereby providing stronger empirical support for the model's adequacy (χ^2^/df = 2.96, CFI = 0.980, RMSEA = 0.053, GFI = 0.984, TLI = 0.966, IFI = 0.984, NFI = 0.976, RFI = 0.950).

**TABLE 2:** Results of model fit indices.

Index	X^2^	P	X^2^/df	CFI	RMSEA
Acceptance range	*p* > 0.05	—	< 3	> 0.9	< 0.1
Original model	95.167	0.001	3.89	0.990	0.089
Revised model	77.004	0.001	2.96	0.980	0.053
Index	GFI	TLI	IFI	NFI	RFI
Acceptance range	> 0.9	> 0.9	> 0.9	> 0.9	> 0.9
Original model	0.998	0.980	0.995	0.989	0.977
Revised model	0.984	0.966	0.984	0.976	0.950

*Note*: Source of Acceptance Range: Hu and Bentler (1999) and Schermelleh‐Engel et al. (2003)

### 3.5 CV and Reliability

To evaluate CV, the CR and the AVE method was used, and the correlation of the AAS with the test anxiety questionnaire, big five personality questionnaire, and BDI was examined. For internal consistency reliability of the AAS, both CR and Cronbach's alpha coefficient were utilized. According to the results, there were significant correlations between the AAS and test anxiety (0.686), neuroticism (0.369), agreeableness (−0.341), conscientiousness (−0.119), extraversion (−0.267), openness to experience (−0.137), and depression (0.755). These results indicate that the AAS has adequate validity (see Table 3).

**TABLE 3:** Mean, standard deviation, and correlation of academic anxiety with test anxiety, personality traits, and depression.

Variables		Mean	Standard deviation	Correlation	Significance level
1	Academic anxiety	18.58	6.91	—	—
2	Test anxiety	40.55	13.32	0.686**	0.001
3	Neuroticism	12.74	2.56	0.369**	0.001
4	Agreeableness	14.69	2.88	−0.341**	0.001
5	Conscientiousness	14.13	2.60	−0.119**	0.001
6	Extraversion	16.88	3.39	−0.267**	0.001
7	Openness to experience	12.38	2.75	−0.137**	0.001
8	Depression	14.23	13.80	0.755**	0.001

*Note*: **p < 0.01, *p < 0.05.

Good internal consistency of the construct is indicated by a CR above 0.60, according to Fornell and Larcker (1981). Additionally, it indicates CV if the average extracted variance is greater than 0.50. The AVE for the AAS, according to the analysis results, was 0.420, which is less than 0.50. Regarding this, Fornell and Larcker (1981) stated that a high CR can make up for a low extracted mean. Additionally, the results showed that the AAS's CR was 0.887 and its Cronbach's alpha coefficient was 0.883, both of which are at an acceptable level and show that the scale has good reliability.

### 3.6 Confirmatory Factor Analysis

**FIGURE 1:**
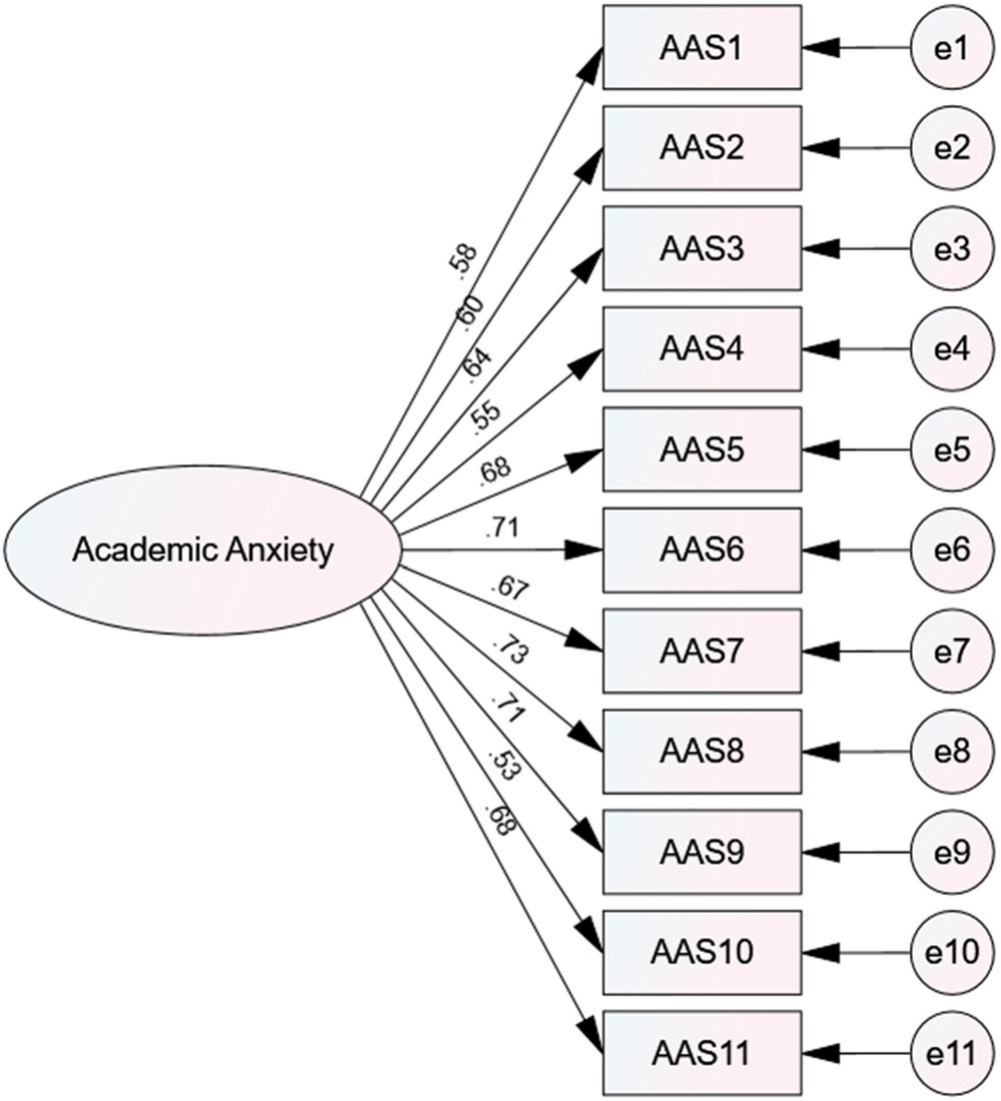
Experimental model of CFA.

In the process of conducting CFA and examining the model fit indices, the results indicated that the initial model did not have an acceptable fit. Therefore, the model was revised, and the covariance between the error terms was established. After this modification, the results of the fit indices are presented in Table 2. Based on this table, the indices RMSEA, χ^2^/df, GFI, CFI, TLI, IFI, NFI, and RFI indicate that the model has a good fit. Figure 1 shows the empirical model of the CFA and the factor loadings of each item on the factor.

## 4 Discussion and Conclusion

The present study aimed to examine the psychometric properties of the AAS in the Iranian population. These results are significant because, on one hand, this research is the first to investigate the statistical characteristics of this scale in an Iranian sample, thereby providing insights into its cross‐cultural applicability. On the other hand, the currently normalized scales for measuring academic anxiety in Iran are based on outdated information and conditions, making the validation of a new scale necessary to keep pace with essential changes.

The results of face validity and content validity indicated that the items of the scale are appropriate for the Iranian community, with no need to remove or modify any of them. The construct validity of the AAS was assessed using CFA with the maximum likelihood method, and CV (AVE > 0.50) was determined and confirmed for this questionnaire. Although the AVE values for some constructs fell slightly below the recommended threshold of 0.50, as suggested by Fornell and Larcker (1981), this may be attributed to the heterogeneity of item content or potential measurement limitations in the AAS. While this may raise concerns regarding CV, it is important to note that the standardized factor loadings for most items exceeded 0.60, and the composite reliability values were acceptable. These findings suggest that despite the low AVE, the constructs still demonstrate reasonable internal consistency and a degree of CV. To assess the reliability and internal consistency of the AAS, composite reliability (CR) and Cronbach's alpha coefficient were used, and the results showed that the scale has good internal consistency and composite reliability.

Finally, to examine concurrent validity, the correlation matrix between the AAS, the test anxiety questionnaire, the big five personality traits, and depression showed that there is a positive and significant relationship between the AAS and test anxiety, neuroticism, and depression, and a negative and significant correlation with agreeableness, conscientiousness, extraversion, and openness to experience. Therefore, the AAS demonstrates good concurrent validity (*p* < 0.01).

In explaining the relationship between test anxiety and academic anxiety, it can be said that test anxiety is a situational form of anxiety that arises specifically during exams and assessments, often stemming from fear of failure or negative evaluation. It constitutes a significant subset of academic anxiety, which encompasses broader concerns and pressures related to academic performance, study planning, and learning abilities. In other words, test anxiety acts as a primary contributor to the development and exacerbation of academic anxiety, since repeated experiences of anxiety during exams can amplify overall academic stress and worry, negatively impacting students' performance. Therefore, managing test anxiety is crucial for mitigating academic anxiety and enhancing academic outcomes (Caviola et al. 2025).

The results obtained regarding the relationship between academic anxiety and depression align with the findings of Nowrozi et al. (2023). Academic anxiety among students manifests as a correlational pattern with mental and behavioral disorders such as depression, conduct disorders, and suicidal tendencies (Bashir et al. 2019). In other words, high levels of academic anxiety increase individuals’ susceptibility to illness, ultimately disrupting their psychological balance and personality health (Ahmadian 2014).

Additionally, the relationship between academic anxiety and the big five personality traits is consistent with studies by Khosravi and Beigdeli (2008) and Asmali (2017). Vosoughi et al. (2012) also showed that neuroticism is a common feature of anxiety and depression disorders and explains the observed comorbidity between these disorders. Furthermore, Hamidi and Bebrazi (2016) found that neuroticism increases academic anxiety.

Personality traits—organized and relatively stable characteristics that differentiate individuals and predict behavior—can influence academic anxiety by affecting its onset or reduction. These traits shape the intensity of anxiety and individuals’ coping mechanisms in response to anxiety‐provoking stimuli (Ahmadian 2014). The relationship between neuroticism and academic anxiety can be explained by the significant impact of neurotic tendencies—characterized by frequent negative emotions such as anxiety, depression, and worry—on the intensity of academic anxiety. Individuals with high neuroticism are generally more sensitive to academic pressures, which exacerbates their anxiety in educational settings. Thus, neuroticism amplifies the severity and frequency of academic anxiety, negatively affecting academic performance and mental health (Nechita et al. 2015). Agreeableness, defined by tendencies toward cooperation, empathy, and adaptability, plays a significant role in mitigating academic anxiety. Highly agreeable individuals tend to form better social interactions in educational environments, benefit from greater social support, and use more effective coping strategies against academic stressors. Consequently, agreeableness reduces the likelihood or severity of academic anxiety and contributes to students’ psychological well‐being (Hayat et al. 2020). Conscientiousness, characterized by responsibility, organization, perseverance, and a goal‐oriented approach, generally helps reduce academic anxiety. Students with high levels of conscientiousness are more likely to manage academic demands effectively through proper planning and time management, which helps prevent anxiety triggered by procrastination or disorganization. Their clear goals and strong internal motivation also contribute to reduced academic stress (Chen et al. 2025). Extraverted individuals, due to their stronger communication skills and wider social networks, tend to handle academic stress more effectively and experience lower levels of anxiety (Ciorbea and Pasarica 2013). Similarly, openness to experience—which includes curiosity, creativity, and receptiveness to new ideas—has a complex but generally positive impact on academic anxiety. Students high in openness often embrace academic challenges and develop innovative coping strategies, which can buffer them against anxiety (Gatzka 2021). s

Taken together, the findings support the AAS as a valid and reliable instrument for assessing academic anxiety in Iranian students. This study offers preliminary but meaningful evidence of the tool's psychometric soundness in the Iranian context.Nonetheless, several limitations should be acknowledged. A primary limitation is the absence of test‐retest data, as reliability was only assessed using internal consistency measures (Cronbach's alpha and composite reliability). Future studies should address this by incorporating test‐retest reliability to strengthen the psychometric evaluation.The innovative aspect of this research lies in its detailed exploration of how specific personality traits, neuroticism, extraversion, conscientiousness, agreeableness, and openness relate to academic anxiety. However, this area remains underexplored, particularly in terms of longitudinal and multidimensional research designs. Much of the existing literature is cross‐sectional and does not sufficiently examine the interactive effects of personality traits, academic anxiety, and contextual factors such as social support, coping mechanisms, and educational environments.Furthermore, future studies should investigate potential gender and cultural differences in these relationships to provide a more nuanced understanding and facilitate the development of practical, culturally sensitive applications of the findings.

Finally, since the sample in this study consisted solely of high school students, the results may not generalize to students at other educational levels. Broader studies across different academic stages are needed to draw more definitive conclusions about the AAS's psychometric properties in the Iranian educational context.

## Author Contributions

**Faezeh Peimanpak**: conceptualization, writingoriginal draft, investigation, funding acquisition, methodology, validation, visualization, writing – review and editing, software, formal analysis, data curation, resources. **Simin Hosseinian**: writing – review and editing, supervision, data curation, conceptualization. **Abbas Abdollahi**: writing – review and editing, supervision, project administration, data curation, methodology.

## Funding

The authors have nothing to report.

## Conflicts of Interest

The authors declare no conflicts of interest.

## Data Availability

The data related to the results of this study are available upon request from the corresponding author. Data is available on Figshare repository at https://figshare.com/s/e474860c879d5d8eb548.
